# The impact of estrogen on periodontal tissue integrity and inflammation—a mini review

**DOI:** 10.3389/fdmed.2025.1455755

**Published:** 2025-02-19

**Authors:** Sucharitha Palanisamy

**Affiliations:** Department of Periodontology and Oral Implantology, SRM Dental College, Chennai, India

**Keywords:** estrogen deficiency, periodontal disease, interlinking mechanisms, tissue integrity, steroid hormones, inflammatory responses

## Abstract

Estrogen is said to be a crucial player in different aspects of periodontal health and disease, influencing a spectrum of cellular and molecular processes within periodontal tissues. Its receptors, ERα and ERβ, are expressed in various periodontal cells, suggesting direct responsiveness to hormonal fluctuations. Mechanistically, estrogen regulates osteoblast and osteoclast activity, thereby affecting bone turnover and maintenance of alveolar bone density. Studies indicate that estrogen upregulates the periodontal ligament stem cells' osteogenic differentiation (PDLSCs), promoting collagen synthesis and matrix mineralization critical for periodontal tissue integrity. Moreover, estrogen's anti-inflammatory properties modulate cytokine expression and immune responses in the periodontium, potentially attenuating periodontal inflammation and tissue destruction. Conversely, estrogen deficiency, such as in postmenopausal women, correlates with increased susceptibility to periodontal diseases characterized by greater clinical attachment loss and alveolar bone resorption. Hormone replacement therapy (HRT) with estrogen has shown promise in clinical settings, demonstrating beneficial effects on periodontal health by reducing inflammation and maintaining alveolar bone density. However, the adequacy and assurance of long-term estrogen supplementation in periodontal management require further investigation due to its systemic effects on other tissues and organs. Understanding the intricate interactions between estrogen and periodontal tissues is crucial for developing targeted therapies that leverage hormonal pathways to enhance periodontal health and mitigate disease progression effectively.

## 1 Introduction

Estrogen is synthesized in two sites which include gonadal (ovaries, placental syncytiotrophoblasts) and extragonadal sites (bone, adipose tissue, liver, adrenal). It plays crucial roles in both “physiological” (normal levels) and “pathophysiological processes” (deficiency) through interactions with estrogen receptors (ERs) (1). These receptors, functioning as ligand-dependent transcription factors, modulate gene expression via estrogen response elements (EREs), thus mediating estrogen's biological effects. There are three types of Estrogen receptors which include ERα, ERβ and GPER1. Of the four recognized forms of estrogen, there are estrone (E1), estradiol (E2), estriol (E3), and estetrol (E4), E2 is the most owing aspect of estrogen to its extensive distribution and significant physiological roles in various tissues and organs. E2 is integral to the development of secondary female sex characteristics, menstrual cycle, and endometrial growth from menarche to menopause, demonstrating high affinity for all the three receptors. E1 is seen more in menopausal women and owes a lower affinity for ERα and ERβ, whereas E3 is produced by the placenta during pregnancy predominantly and shows a marked increase during this period but remains negligible otherwise. E4 is a fetal estrogen detectable only during pregnancy and has an even lower affinity for ERs than estradiol (2). Estrogen is essential for periodontal health since it regulates the inflammatory response, affects physiological factors such periodontal cell proliferation and differentiation, and maintains alveolar bone density. It exerts its effects by binding to estrogen receptors (ERα & ERβ) in periodontal tissues, thereby regulating gene expression associated with inflammation and bone metabolism. Tissue responses to hormonal variations during puberty, pregnancy, and menopause are not uniform, as each stage presents distinct hormonal profiles and biological demands that elicit varied effects on periodontal tissues (3). During puberty, elevated estrogen and progesterone increase vascular permeability and inflammatory sensitivity, leading to transient gingival changes such as puberty gingivitis. In pregnancy, surges in progesterone and estrogen amplify inflammatory responses to plaque and promote angiogenesis, often resulting in pregnancy gingivitis or pyogenic granulomas, driven by a Th2-dominant immune profile. Conversely, menopause is marked by a decline in estrogen, causing atrophic changes, impaired collagen synthesis, and alveolar bone resorption, exacerbated by a shift towards a pro-inflammatory Th1 and Th17 cytokine profile (4). These stage-specific responses reflect the dynamic interplay between hormonal levels, receptor sensitivity, and local tissue adaptation, underscoring the unique nature of biological responses at each phase. Estrogen deficiency, particularly during menopause, has been linked to increased susceptibility to periodontal disease due to enhanced pro-inflammatory cytokine production and decreased bone mineral density, leading to alveolar bone resorption. Moreover, the significance of estrogen in sustaining periodontal health is highlighted by its function in keeping the structural integrity of both the gingival epithelium & connective tissue. This mini-review aims to explore the role of estrogen on periodontal health by emphasizing its influence on periodontal tissue homeostasis and inflammatory processes, while also offering valuable insights into its clinical significance and potential therapeutic applications within the field of periodontics.

## 2 Activation pathways triggered by estrogen receptors (ER) in the periodontium

**Figure 1:**
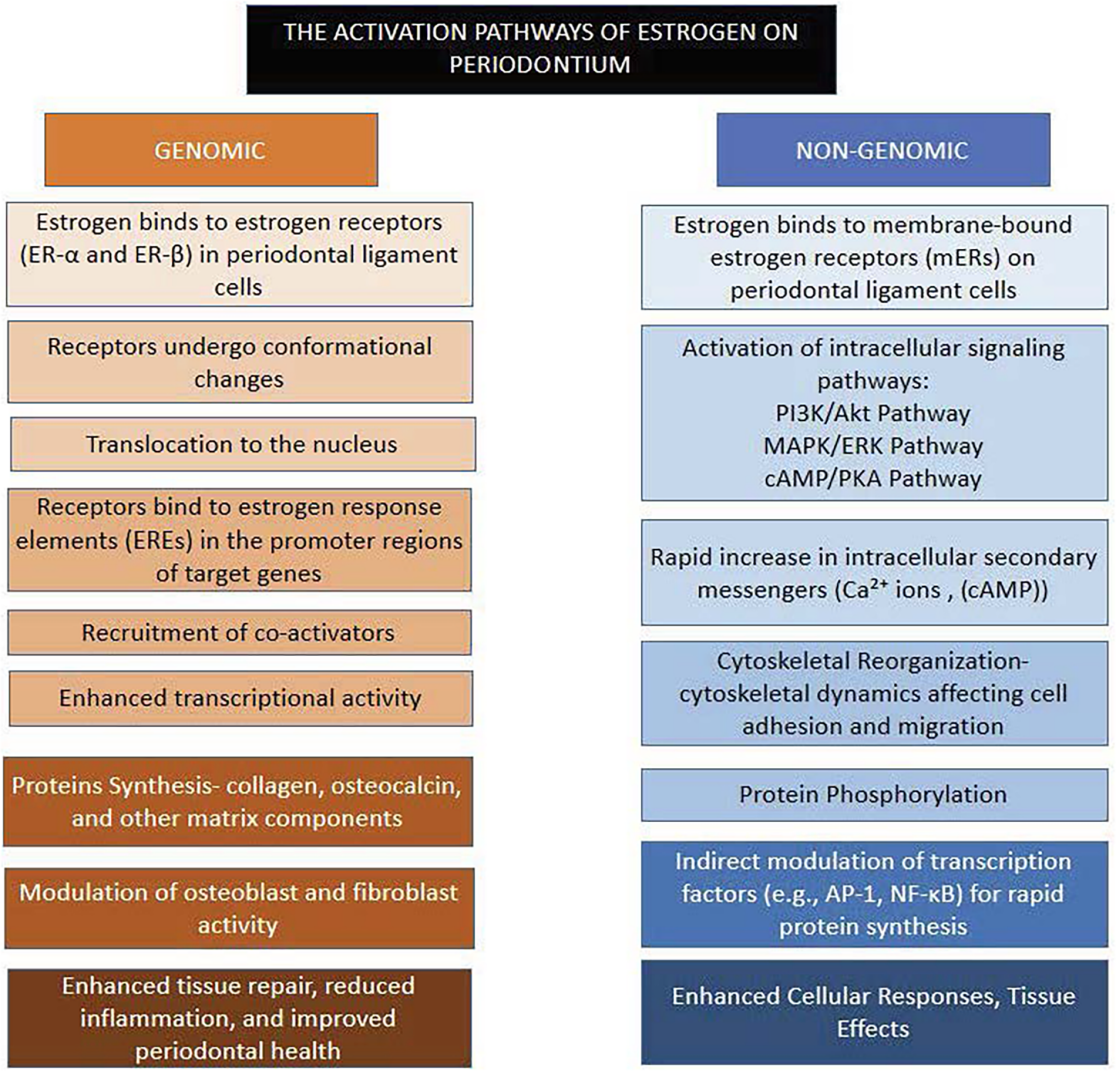
Activation pathways triggered by estrogen receptors (ER) in the periodontium.

The mechanisms of ER-mediated signaling can be classified into genomic and non-genomic pathways, each contributing to periodontal homeostasis and repair (Figure 1).

### 2.1 Genomic pathways

The genomic pathway, often referred to as the classical estrogen signaling mechanism, involves the direct regulation of gene expression. Estrogen, being lipophilic, diffuses through the plasma membrane and binds to intracellular ERα or ERβ, leading to receptor dimerization. These dimers translocate to the nucleus, where they bind to estrogen response elements (EREs) in the promoter regions of target genes (5). This interaction recruits coactivators or corepressors to modulate transcription. In the gingival tissues, this pathway enhances the synthesis of extracellular matrix (ECM) components such as collagen and fibronectin, ensuring structural integrity and resilience. In alveolar bone, estrogen upregulates the expression of osteoprotegerin (OPG), a decoy receptor that inhibits receptor activator of nuclear factor kappa-B ligand (RANKL) (6). This action prevents osteoclastogenesis and mitigates bone resorption. In immune cells, estrogen dampens pro-inflammatory cytokine production (e.g., IL-1β and TNF-α) and promotes anti-inflammatory cytokines (e.g., IL-10), contributing to an immunoregulatory environment in periodontal tissues.

### 2.2 Non-genomic pathways

Non-genomic signaling mediated by membrane-bound ERs and GPER1 elicits rapid cellular responses independent of direct transcriptional regulation. In Mitogen-Activated Protein Kinase (MAPK) Pathway, estrogen activates ERK1/2 and p38 MAPK, promoting cellular proliferation, migration, and differentiation in periodontal ligament fibroblasts and osteoblasts (7). This pathway enhances ECM turnover and tissue remodeling, crucial for maintaining periodontal architecture. In Phosphatidylinositol-3 Kinase (PI3K)/Akt Pathway, estrogen stimulates PI3K, leading to Akt activation, which supports cell survival by inhibiting pro-apoptotic factors and promoting anti-apoptotic signals. In alveolar bone, this pathway enhances osteoblast viability and activity, counteracting bone loss (8). In Calcium and Nitric Oxide Signaling, estrogen induces calcium influx and activates endothelial nitric oxide synthase (eNOS), leading to nitric oxide (NO) production. NO facilitates vasodilation and angiogenesis, improving vascularization and nutrient delivery in periodontal tissues (9).

## 3 The role of estrogen in bone, salivary gland, gingiva and periodontal ligament

Estrogen plays a crucial role in maintaining the health and function of various tissues, including bone, gingiva, periodontal ligament, and salivary glands. In bone, estrogen promotes osteoblast activity and inhibits osteoclast-mediated resorption, thereby preserving bone density and strength. Within the gingiva and periodontal ligament, estrogen supports cellular proliferation, differentiation, and collagen synthesis, essential for maintaining the structural integrity and resilience of periodontal tissues. Additionally, estrogen influences the function of salivary glands, enhancing saliva production and secretion, which is vital for oral health, lubrication, and antimicrobial defense. The hormone's regulatory effects on these tissues underscore its importance in oral and skeletal health, with deficiencies potentially leading to conditions such as osteoporosis, periodontal disease, and xerostomia.

### 3.1 Estrogen role in bone homeostasis

Bone resorption decreases and the self-regeneration of early mesenchymal progenitors is suppressed due to estrogen's three principal effects on bone metabolism. Firstly, estrogen inhibits the initiation of bone remodeling and the formation of new basic multicellular units (BMUs). Secondly, it limits osteoclast differentiation and promotes their apoptosis. Lastly, estrogen supports the adhesion and differentiation of osteoblastic cells while preventing their apoptosis, thereby maintaining bone formation at the cellular level. It is a crucial regulator of bone metabolism, significantly impacting skeletal growth and homeostasis in both genders. Estrogen acts directly on the bone cells (osteoblasts, osteocytes, immune cells) through estrogen receptors (ERs) present on these cells (10, 11).

#### 3.1.1 Osteocytes

Estrogen plays a pivotal role in maintaining osteocyte viability and function, crucial for skeletal homeostasis. It exerts its effects by binding to estrogen receptors on osteocytes, regulating the expression of genes involved in bone remodeling and apoptosis. Estrogen inhibits osteocyte-mediated bone resorption by suppressing the production of receptor activator of nuclear factor kappa-Β ligand (RANKL) and promoting osteoprotegerin (OPG) synthesis, thus balancing osteoclast activity. Furthermore, estrogen mitigates oxidative stress and inflammatory cytokines within the bone microenvironment, preserving osteocyte integrity and preventing excessive bone turnover. Its deficiency, as seen in postmenopausal women, disrupts these mechanisms, leading to osteocyte apoptosis, compromised bone matrix, and increased risk of osteoporosis.

#### 3.1.2 Osteoblasts

The estrogen receptors alpha (ERα) and beta (ERβ) on osteoblasts modulates gene expression that promotes cellular proliferation, differentiation, and matrix mineralization (12). Estrogen enhances the production of type I collagen and osteocalcin, critical components of the bone matrix, while suppressing pro-apoptotic pathways, thereby prolonging osteoblast viability. Additionally, it inhibits the synthesis of inflammatory cytokines like IL-6 and TNF-α, which otherwise impair osteoblast activity and promote bone resorption. The absence of estrogen, as seen in postmenopausal osteoporosis, results in reduced osteoblast function, leading to impaired bone formation and structural deterioration.

#### 3.1.3 Osteoclasts

Estrogen directly suppresses osteoclastogenesis by reducing the expression of receptor activator of nuclear factor kappa-Β ligand (RANKL) and increasing osteoprotegerin (OPG) production by osteoblasts and stromal cells, thereby inhibiting RANKL-mediated osteoclast differentiation (13). Estrogen also induces osteoclast apoptosis through caspase activation while attenuating the expression of pro-resorptive cytokines such as IL-1, IL-6, and TNF-α. Furthermore, it stabilizes the bone microenvironment by reducing oxidative stress and inflammatory mediators, which would otherwise enhance osteoclast survival and function (14, 15).

Activation of ER signaling pathways generally promotes osteoblast differentiation and suppresses osteoclastic activity. Additionally, estrogen prevents osteocyte apoptosis, with its anti-apoptotic effects linked to the regulation of autophagy in these cells. Estrogen insufficiency leads to increased osteoclastogenesis, extended osteoclast lifespan, and upregulated bone turnover, resulting in accelerated bone resorption (16). Research indicates in osteocytes, estrogen shortage decreases autophagy and promotes apoptosis, while estrogen replacement therapy boosts osteocyte viability by preventing apoptosis and preserving autophagy (17). Furthermore, to its immediate impact on bone cells, estrogen influences immune response and oxidative stress, which in turn governs bone homeostasis. Aromatase enzyme activity is observed in osteoblasts, chondrocytes, and fibroblasts, where these enzymes play a crucial role in converting androgens into estrogens. During puberty, bone growth slows down owing to the estradiol (E2) produced locally, which promotes early epiphyseal advancement. The peak activity of the hypothalamic gonadotropic axis produces elevated levels of E2, leading to a spike in chondrocyte apoptosis in the epiphyseal plate, thereby decelerating ossification and growth. Estrogen enhances osteoblast survival and activity through ER alpha receptors present on osteoblast cells (18). Additionally, estrogen decreases the RANKL-OPG axis, thereby reducing osteoclastogenesis as well as differentiation of osteocytes to osteoclasts. Pro-inflammatory cytokines, such as prostaglandin E2, granulocyte macrophage colony-stimulating factor, IL-1, IL-6, and TNF alpha, are produced when estrogen levels are lowered which in turn stimulate osteoclastic activity thereby significantly influencing bone metabolism by enhancing bone resorption (19). TNF-α, IL-6, and IL-1 in particular are known to be osteoclastogenic cytokines that promote bone resorption. Insufficient estrogen triggers inflammatory pathways, which raise M-CSF and RANKL synthesis (20). M-CSF binds to its receptor, promoting osteoclast proliferation and the survival of osteoclast precursors and mature osteoclasts. Similarly, RANKL binds to RANK receptors, stimulating osteoclast differentiation and activity while preventing their apoptosis (21). Women exhibit a higher prevalence of oral diseases, such as periodontal diseases and temporomandibular joint disorder (TMD), suggesting a significant role of estrogen signaling in these pathologies. Fluctuating estrogen levels during the childbearing years can exacerbate facial pain, while elevated estrogen levels during pregnancy promote gingivitis. Conversely, reduced estrogen levels during menopause are associated with increased susceptibility to temporomandibular joint degeneration and heightened alveolar bone loss (22). In 12-week-old female mice, but not in males, the deletion of ERα, which is selectively expressed in mature osteoclasts and controlled by the cathepsine K (CtsK) promoter, was linked to trabecular bone loss (23). Ovariectomy resulted in minor additional bone loss, while E2 treatment restored trabecular bone mass without affecting the cortical compartment (24). A greater percentage of osteoclasts and enhanced bone turnover in trabecular bone were connected to a loss in trabecular bone mass in CtsK-Cre + Erαlox/lox mice. Similar results were obtained using a second model that concentrated on myeloid osteoclast precursors via the lysozyme M (LysM) promoter. Trabecular bone mass and microarchitecture were changed in mutated female mice due to an increased number of osteoclast progenitors in the bone marrow and differentiated osteoclasts in the vertebrae, but cortical bone was unchanged (24–26). This study investigated the effects of estrogen on the bone regeneration capacity of periodontal ligament stem cells (PDLSCs) derived from osteoporotic rats and cultured on a collagen-based composite scaffold. Two groups of forty-eight 3-month-old, healthy Sprague-Dawley female rats were assembled: rats that had bilateral ovariectomies (OVX) and rats that had sham operations. The OVX group's cells' ability to proliferate was greatly decreased upon treatment with 17β-estradiol (E2), but their osteogenic differentiation was improved and their levels of ALP, OCN, ERα, and ERβ mRNA expression were raised (27). Estrogen deficiency leads to alterations in the microarchitecture of maxillary alveolar bone. In long bones, estrogen primarily acts through estrogen receptor α (ERα), which is essential for preserving the microarchitecture of maxillary alveolar bone. This involves controlling the differentiation and mortality of bone cells in addition to affecting the local synthesis of inflammatory cytokines like IL-33, TNF-α, and IL-1β (28). Estrogens are crucial for bone development, maturation, and bone remodeling in adults. Whereas the implications of 17β-estradiol (E2) on long bones and vertebrae have been thoroughly explored, its actions in the mandible remain less understood but are potentially significant for therapeutic strategies aimed at addressing bone loss (29). Tooth loss and the subsequent resorption of alveolar ridges represent notable oral health challenges in elderly individuals. Various factors contribute to tooth loss, and several studies have identified a correlation between tooth loss, residual ridge resorption, and systemic osteoporosis. Investigations into estrogen replacement therapy (ERT) have explored its potential benefits in mitigating these conditions, revealing modest advantages associated with ERT (30).

### 3.2 Estrogen role in salivary gland

**Figure 2:**
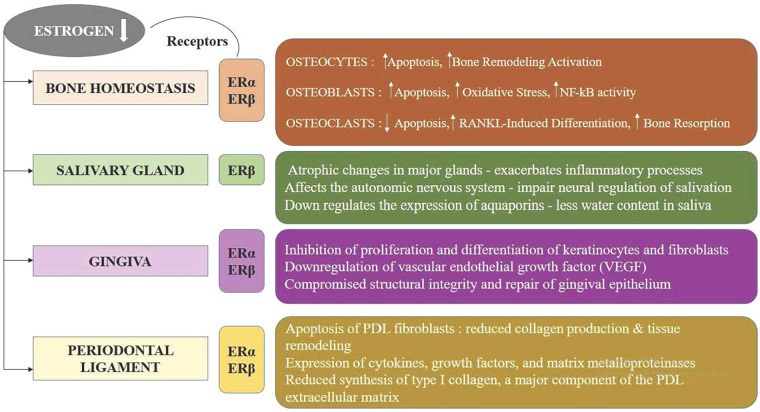
The impact of estrogen deficiency on bone, salivary glands, gingiva, and periodontal ligament.

Estrogen is essential for maintaining the structural and functional integrity of salivary glands. These glands possess atrophic alterations as a result of the lowering in estrogen levels postmenopausal, diminishing their saliva production and secretion capabilities (Figure 2) (31). Additionally, estrogen affects the autonomic nervous system, which controls salivary gland function; its deficiency can impair neural regulation of salivation. Estrogen also regulates the expression of aquaporins, proteins facilitating water transport in salivary glands, and reduced estrogen levels decrease this water transport, leading to lower saliva flow. Moreover, estrogen deficiency exacerbates inflammatory processes within the salivary glands, further impairing their function and promoting xerostomia (32, 33). Consequently, postmenopausal women frequently experience significant reductions in salivary flow and alterations in saliva composition, resulting in dry mouth symptoms and an increased risk of periodontal disease progression, dental caries, mucosal infections, and difficulties in swallowing and speaking (34). Reduced levels of E3 during menopause affects oral epithelial maturation, resulting in delicate, atrophic epithelium prone to inflammatory changes. Similarly, immune cells and salivary gland epithelial cells both demonstrate estrogen receptors alongside are known to inflect epithelial maturation in classic target organs (35). Estrogen deficiency significantly reduces saliva production by inducing atrophic changes in the salivary glands and impairing neural regulation of salivation. This reduction in salivary flow, or xerostomia, diminishes the protective functions of saliva, such as its antimicrobial properties and its role in maintaining oral pH balance (36, 37). Consequently, the oral environment becomes more susceptible to bacterial colonization and plaque formation, increasing the risk of periodontal disease. The decreased saliva also exacerbates inflammatory processes in periodontal tissues, further contributing to the progressive destruction and intensity of periodontal disease in postmenopausal women. Hormone replacement therapy (HRT) has been shown in numerous studies to reduce oral discomfort in postmenopausal women, highlighting the function of female sex hormones in preserving oral tissues (38, 34).

### 3.3 Estrogen role in gingiva

It has been shown that sex steroid hormones affect target tissues' cellular growth, differentiation, and proliferation both directly and indirectly. This includes fibroblasts and gingival keratinocytes. The actions of these hormones on these cells are explained by two basic theories: first, they affect collagen maintenance and repair, and second, they change the efficiency of the epithelial barrier against bacterial infection (39). Healthy premenopausal gingival fibroblast cells can exhibit both reduced protein synthesis and cellular proliferation upon exposure to estradiol (40). This growth appears to be caused by a particular cell subpopulation in the parent culture that reacts to physiological levels of estrogen. Estrogen receptors (ERα and ERβ) are present in gingival fibroblasts, osteoblasts, and immune cells, enabling estrogen to modulate local inflammatory responses, collagen synthesis, and bone metabolism. In the gingiva, estrogen promotes the proliferation and differentiation of keratinocytes and fibroblasts, which are crucial for maintaining the structural integrity and repair of gingival epithelium and connective tissue. Estrogen's anti-inflammatory properties are particularly significant in gingival tissue, because it stimulates the generation of the anti-inflammatory cytokine (interleukin-10) and downregulates the pro-inflammatory cytokines such as interleukin-1β, tumor necrosis factor-alpha and interleukin-6. This modulation maintains a balanced immune response, reducing the risk of chronic inflammation and periodontal disease. Furthermore, estrogen influences angiogenesis by upregulating vascular endothelial growth factor (VEGF), which in turn affects gingival vascularization. This enhances blood supply, delivering essential nutrients and immune cells to the gingival tissues, thereby supporting tissue repair and resilience against bacterial insults. In postmenopausal women, decreased estrogen levels are associated with increased gingival inflammation, a higher prevalence of periodontal disease, and reduced bone density, exacerbating alveolar bone loss. Estrogen also regulates the periodontal ligament (PDL) by influencing the turnover and homeostasis of collagen and other extracellular matrix components. It enhances the expression of collagen types I and III, which are essential for PDL strength and flexibility. Additionally, estrogen modulates the activity of matrix metalloproteinases (MMPs), enzymes involved in tissue remodeling, thereby preventing excessive degradation of the PDL. An increase in ovarian hormones, specifically estrogen and progesterone, heightens the risk of gingival inflammation. Pregnancy and the administration of oral contraceptives (OCs) cause this rise, both of which elevate hormone levels. Elevated hormone levels promote bacterial proliferation in the oral cavity, leading to a shift in the bacterial flora. Progesterone and estrogen stimulate important inflammatory response components. Progesterone modifies the gingiva's pace and rhythm of collagen synthesis, impairing its ability to repair and maintain itself. Furthermore, progesterone and estrogens contribute to folate deficiency, further hindering gingival repair, and suppress the immune system (41). A research investigation explored the significance of estrogen receptor β (ERβ) in oral mucosal cells and the suggested mechanistic causal connection among cell behavior and low-dose BPA-modulated calcium ion (Ca2+) influx (42). The host response plays a crucial aspect in periodontal disease, with mast cells being particularly significant. These cells appear to be impacted by estrogen deficiency. The purpose of the study was to assess mast cells and metalloproteinase (MMP)-9 expression in rats with ovariectomized alongside with gingival inflammation. The findings concluded that periodontitis resulted in a mast cell downregulation and a contrast in MMP-9 expression. Ovariectomy alone did not change MMP-9 expression or affect mast cell presence in the rat papilla, but when combined with inflammation, it led to a reduction in mast cells (43). A study investigated the dynamics of glycosaminoglycans (GAGs) in rat gingiva and the impact of sex steroid deficiency, as well as hormonal therapies involving estrogen and dexamethasone (DEX). Estradiol appeared to influence GAG levels, and DEX did not counteract estradiol's effects on gingival GAGs. The histotypical structure of gingiva correlates with chondroitin sulfate levels, suggesting that estrogen therapy could play a crucial aspect in maintaining gingival health (44).

### 3.4 Estrogen level in periodontal ligament

The female hormone estrogen is essential for preserving both the functional integrity and homeostasis of the periodontal apparatus. At the cellular level, estrogen influences the proliferation, differentiation, and apoptosis of PDL fibroblasts, which are essential for collagen production and tissue remodeling. It modulates the expression of cytokines, growth factors, and matrix metalloproteinases, thereby regulating the inflammatory response and matrix turnover within the PDL. Additionally, Type I collagen formation is aided by estrogen, which is a crucial part of the extracellular matrix of PDL, and supports angiogenesis, ensuring an adequate blood supply to the tissue (22). Furthermore, PDL cells express ERα and ERβ, a pair of estrogen receptors, mediating estrogen's genomic and non-genomic actions. This hormonal regulation is particularly significant in the context of periodontal health, as fluctuations in estrogen levels, such as those occurring during menopause, pregnancy, or hormone replacement therapy, can impact PDL structure and function, potentially contributing to increased susceptibility to periodontal diseases. A study examined how estrogen affected the expression of osteoprotegerin (OPG) and alkaline phosphatase (ALP) within human periodontal ligament cells (hPDLCs). The findings suggest that estrogen may promote periodontal health and exert antiresorptive effects by enhancing the expression of ALP and OPG in hPDLCs (45). Numerous studies focused on delineating the expressive patterns of estrogen receptor (ER) in PDL cells and to explore their functional significance. Human PDL cells exhibited immunoreactivity for ERβ but not ERα, indicating that the impacts of estrogen in these cells are mediated through ERβ. Additionally, the lack of immunoreactivity for progesterone receptors in PDL cells suggests that progesterone has no direct influence on PDL cell function (46, 47, 48, 49). The study focused on the role of estrogen by identifying estrogen receptors in the pulpal tissue and periodontal ligament apparatus, as well as the histological alterations in the maxillary sinus and surrounding dental tissues during pregnancy. The study found that during pregnancy, there were insignificant inflammatory alterations and elevated concentrations of ERα in the oral and nasal mucosa tissues (50). An investigation was conducted into the function of the Wnt/β-catenin signaling pathway in the osteogenic development associated with human periodontal ligament stem cells (hPDLSCs) that is boosted by estrogen. According to the study, hPDLSCs' osteogenic differentiation is enhanced by estrogen (E2), which is mediated through the stimulation of the Wnt/β-catenin signaling pathway, which involves Wnt3a particularly (51). Osteoporotic women often experience significant bone loss and compromised bone density. Estrogen insufficiency, a crucial element in the onset of osteoporosis after menopause, has gained considerable limelight in periodontal disease research. Both ER receptors are expressed in PDLCs. PDLCs exhibited elevated osteocalcin production and alkaline phosphatase (ALP) function with exposure to 17-beta estradiol, underscoring the function of estrogen in promoting periodontal health and regulating periodontal disease (52, 53). The production of receptor activator of nuclear factor-kappa B ligand (RANKL) and its corresponding decoy receptor, osteoprotegerin (OPG), by periodontal ligament (PDL) cells, which control osteoclastogenesis, can be influenced by estrogen. Treatment with estradiol increased OPG expression and decreased RANKL expression in human PDL cells, indicating an antiresorptive effect (54). Moreover, estrogen (E2) significantly increases mesenchymal stem cells' capacity for proliferation and osteogenic differentiation while maintaining the embryonic nature of human periodontal ligament stem cells (hPDLSCs) across long-term culture. These effects suggest promising implications for its utilization in tissue engineering applications (55). Estrogens and their receptors are critical in maintenance of periodontal ligament (PDL) tissue. Proline-, glutamic acid-, and leucine-rich protein 1 (PELP1), a regulator of estrogen receptors (ER), likely plays a role in alveolar bone formation and periodontal ligament (PDL) equilibrium. Studies investigating PELP1 expression in rat PDL tissue under different estrogen levels observed a significant upregulation of PELP1 expression following estrogen treatment (56). Osteoporosis has been linked to increased clinical attachment loss in individuals with periodontitis. Experimental research has demonstrated promising outcomes in treating osteoporosis using pulsed electromagnetic field (PEMF) stimulation. Application of PEMF appears to mitigate bone loss effects observed in cases of periodontitis and ovariectomy (57).

### 3.5 Estrogen level in immune cells

Estrogen exerts multifaceted immunomodulatory effects on individual immune cells through its interaction with estrogen receptors (ERα, ERβ, and GPER1), which activate genomic and non-genomic signaling pathways. In innate immunity, macrophages exhibit a dose-dependent response, with low estrogen levels promoting a pro-inflammatory M1 phenotype, enhancing cytokine production (e.g., IL-1β, TNF-α) and microbial defense, while higher levels favor an anti-inflammatory M2 phenotype characterized by IL-10 and TGF-β secretion, essential for tissue repair and resolution of inflammation (58). Dendritic cells, crucial for antigen presentation, show increased maturation and expression of co-stimulatory molecules like CD80 and CD86 under estrogenic influence, enhancing T-cell activation while modulating their cytokine output to maintain immune homeostasis. Estrogen also tempers neutrophil activity by regulating chemotaxis, reactive oxygen species production, and neutrophil extracellular trap (NET) formation, reducing excessive tissue damage (59). In natural killer (NK) cells, estrogen downregulates activating receptors like NKG2D, diminishing cytotoxic activity to support immune tolerance. In adaptive immunity, estrogen shifts T-cell responses toward a T helper 2 (Th2) phenotype by promoting IL-4 and IL-13 production while suppressing T helper 1 (Th1) and T helper 17 (Th17) pathways, reducing inflammatory cytokines such as IFN-γ and IL-17. It also enhances regulatory T cell (Treg) differentiation, ensuring immune tolerance and control of excessive inflammatory responses. Estrogen influences B lymphocytes by increasing their survival, proliferation, and antibody production, while also contributing to the generation of autoantibodies in autoimmune conditions. In the periodontium, these effects are particularly relevant during hormonal fluctuations, such as during pregnancy or menopause. Elevated estrogen levels during pregnancy promote vascularization and a Th2-dominant immune response, often resulting in pregnancy-associated gingivitis, whereas postmenopausal estrogen deficiency leads to a pro-inflammatory state, heightening the risk of periodontitis and alveolar bone loss. These regulatory effects underscore estrogen's critical role in maintaining immune equilibrium, modulating inflammatory responses, and influencing periodontal health.

## 4 Clinical approaches and perspectives for managing periodontal patients with fluctuating estrogen levels

Managing periodontal patients with fluctuating estrogen levels requires a nuanced, multifaceted clinical approach, recognizing the impact of hormonal variations on periodontal tissue homeostasis, immune response, and inflammatory pathways (60). During puberty, where hormonal surges can heighten gingival inflammation, clinicians should emphasize preventive care, focusing on meticulous oral hygiene practices and regular periodontal check-ups. Early detection of puberty-related gingivitis, often characterized by gingival hyperemia and edema, allows for timely intervention and management of plaque-induced inflammation (61). In addition, educating patients about the hormonal influences on periodontal health can foster better self-care practices, particularly in the context of increased plaque accumulation during this developmental stage. In pregnant patients, heightened progesterone and estrogen levels can lead to exaggerated inflammatory responses, manifesting as pregnancy gingivitis or pyogenic granulomas. Regular monitoring of gingival health is crucial, with adjustments to scaling and root planing techniques to minimize gingival irritation (62). The use of antimicrobial agents, such as chlorhexidine, is often considered safe for controlling plaque buildup and preventing further exacerbation of inflammation (63). Pregnancy-related hormone-induced inflammation should be distinguished from periodontal disease, as the former is typically reversible postpartum. However, clinicians should be vigilant for potential systemic effects of untreated periodontal disease, such as the risk of preterm birth, and manage the condition accordingly. For postmenopausal patients, the decline in estrogen levels contributes to reduced collagen synthesis, impaired bone metabolism, and an increased risk of periodontal bone loss (64, 65). The clinical management of these patients should focus on comprehensive periodontal therapy, including scaling and root planing, with an emphasis on preserving alveolar bone density. Hormone replacement therapy (HRT) may be considered in some cases to mitigate bone resorption and support tissue integrity, but its application must be carefully assessed, weighing the potential benefits against risks such as an increased risk of oral cancer or cardiovascular conditions (66, 67, 68). Additionally, non-hormonal treatments, such as bisphosphonates or selective estrogen receptor modulators (SERMs), may be explored for enhancing bone health in postmenopausal women. Monitoring inflammatory markers, cytokine levels, and biomarkers of bone turnover can offer additional insights into the patient's periodontal health status and guide more targeted interventions. Furthermore, interdisciplinary collaboration with endocrinologists may be essential for optimizing hormonal management and minimizing systemic effects on periodontal tissues.

## 5 Conclusion

Estrogen plays a multifaceted role in periodontal health and disease, influencing various cellular and molecular pathways within the periodontal tissues. It stimulates periodontal ligament stem cells to differentiate into osteogenic forms and enhances the synthesis of extracellular matrix components crucial for tissue integrity. Estrogen's modulation of inflammatory mediators and its antiresorptive effects through the regulation of RANKL/OPG ratio contribute significantly to maintaining periodontal homeostasis. Moreover, estrogen receptors (ERα and ERβ) expressed in periodontal tissues suggest a direct responsiveness to hormonal fluctuations, such as those occurring during menopause or hormone replacement therapy, which may impact periodontal health outcomes. Future research should delve deeper into understanding the specific mechanisms by which estrogen influences periodontal tissue remodeling, immune responses, and vascularization, thereby paving the way for targeted therapeutic interventions aimed at enhancing periodontal health in both physiological and pathological conditions.
